# Bie Jia Jian pill enhances the amelioration of bone mesenchymal stem cells on hepatocellular carcinoma progression

**DOI:** 10.1007/s11418-021-01548-4

**Published:** 2021-07-23

**Authors:** Huang Jingjing, Huang Hongna, Wang Xiaojiao, Guo Yan, Zhong Yuexue, Hu Yueqiang

**Affiliations:** 1 Department of Spleen, Stomach and Liver Diseases, The First Affiliated Hospital of Guangxi University of Chinese Medicine, Nanning, P.R. China; 2 Guangxi Key Laboratory of Translational Medicine for Treating High-Incidence Infectious Diseases With Integrative Medicine, Nanning, P.R. China; 3 Teaching and Research Office of Internal Medicine of Traditional Chinese Medicine, The First Affiliated Hospital of Guangxi University of Chinese Medicine, No. 89-9, Dongge Road, Qingxiu District, Nanning, 530200 Guangxi P.R. China; 4 The First Clinical Medical College of Guangxi University of Chinese Medicine, Nanning, P.R. China; 5 Guangxi Key Laboratory of Basic Research of Traditional Chinese Medicine, Nanning, P.R. China

**Keywords:** Hepatocellular carcinoma, Cancer stem cells, Bie Jia Jian pill, MicroRNA 140, Bone mesenchymal stem cells

## Abstract

**Background:**

The therapeutic efficiency of Traditional Chinese Medicine (TCM) in suppressing the recurrence and metastasis of hepatocellular carcinoma (HCC) has been well proved.

**Objective:**

The aim of this study is to investigate the role of Bie Jia Jian pill (BJJP) combined with bone mesenchymal stem cells (BMSCs) in HCC progression.

**Methods:**

Flow cytometry was used to identify BMSCs isolated from BALB/c mice. The expressions of biomarkers and apoptosis rate of cancer stem cells (CSCs) enriched from Huh7 cells were also measured. The osteogenic differentiation and adipogenic differentiation ability of isolated BMSCs was determined by oil red O staining and Alizarin Red Staining. CSCs were used to establish the orthotopic HCC model. Histological changes in the liver tissues were examined by hematoxylin–eosin (H&E) staining and Van Gieson (VG) staining. The cell apoptotic rate in the cancer tissues was detected by TUNEL staining. The cell proliferation antigen Ki67 in the cancer tissues were detected by immunofluorescence assay and PCR, respectively. The levels of CSCs cellular surface markers (CD24, CD133 and EpCAM) and Wnt/β-catenin signal pathway related proteins were detected by PCR and western blot.

**Results:**

Treatment of BJJP or BMSCs both improved the morphology induced by HCC and suppressed the differentiation ability of CSCs, as evidenced by down-regulated expressions of CD24, CD133, EpCAM and Ki67. The protective effect of BJJP or BMSCs in cancer tissues can be enhanced by the combination of BJJP and BMSCs. In addition to that, BJJP or BMSCs alone was found to increase the expression of miR-140 and promote cell apoptosis in CSCs, while down-regulation of miR-140 partially reversed the protective effect of BMSCs or BJJP + BMSCs on cancer tissues. BJJP + BMSCs treatment together also can down-regulate the expressions of Wnt3a and β-catenin.

**Conclusions:**

These results proved the inhibitory role of BJJP + BMSCs in HCC development through regulating miR-140 and Wnt/β-catenin signal pathway.

**Supplementary Information:**

The online version contains supplementary material available at 10.1007/s11418-021-01548-4.

## Introduction

Primary liver cancer represents one of the leading causes of cancer-associated mortalities globally [1]. Hepatocellular carcinoma (HCC) comprises about 85–90% of primary liver cancer with the highest occurrence in Asia and Sub-Saharan Africa [2, 3]. HCC is also the main type of adult primary liver cancer, characterized by high recurrence rate and poor prognosis in China [4]. Although great progress has been made in the diagnosis and treatment of HCC, the 5-year survival rate remains far from satisfactory.

Mounting evidence shows that tumors may develop from a minor subpopulation of cancer cells with stem-like characteristics, which are defined as cancer stem cells (CSCs) [5]. Mesenchymal stem cells (MSCs) are multi-potent cells with the capacity of self-renewal and differentiation [6]. Hepatocyte-like cells with hepatocyte function can be induced from MSCs [7]. Bone mesenchymal stem cells (BMSCs), originating from bone marrow, play a crucial role in tissue maintenance and regeneration, and are the most widely studied stem cell source in liver tissue engineering [8].

MicroRNAs (miRNAs) are the small non-coding RNAs with 20–25 nucleotides in length. MiRNAs can affect a variety of cellular functions, such as cell differentiation, proliferation and apoptosis [9]. Aberrant expressions of miRNAs have been demonstrated to implicate in cancer initiation and progression in many cancers, including HCC [10]. MicroRNA 140 (miR-140) is discovered as a down-regulated miRNA in HCC [11]. Yang et al. demonstrate that miR-140-5p is a tumor suppressor gene in HCC and an important biomarker for HCC prognosis [12].

Recent studies suggest that Traditional Chinese Medicine (TCM) plays an important role in the entire process of cancer prevention and treatment [13]. TCM as a kind of adjuvant therapy contributes to the overall survival of patients with HCC [14]. Bie Jia Jian pill (BJJP, the ingredient of BJJP includes: soft-shelled Turtle shell glue, Ass-hide Gelatin, Honeycomb, Pillbug Armadillidium, Ground Beetle, dung beetle, saltpeter, Chinese Thorowax Root, baical skullcap root, Pinellia Ternata, MedicinaI Changium Root, Dried Ginger, officinal magnolia bark, Ramulus Cinnamomi, White Paeony Root, Blackberrylily Rhizome, Peach Seed, Tree Peony Bark, rhubarb root and rhizome, Chinese trumpet creeper, Pepperweed Seed, Japanese Felt Fern, dianthus superbus, as showed in the website of China National Pharmaceutical Group Co., Ltd.) is a kind of TCM from "the Synopsis of Prescription of the Golden Chamber" with satisfactory therapeutic achievement in improving liver fibrosis [15]. However, the protective role of BJJP in HCC is still unknown. In this study, we established the orthotopic HCC model to investigate the possible interaction of BJJP and BMSCs in HCC progression.

## Materials and methods

### Animals

Male BALB/c mice were provided by the Hubei Provincial Laboratory Animal Public Service Center (Wuhan, China). Male BALB/c nude mice (body weight, 18 ± 2 g) were provided by Charles River Labs (Beijing, China). All animals were housed in the specific pathogen free (SPF) leveled animal room under a 12–12 h light/dark cycle at 24–26 °C with free access to food and water. All experiments were conducted in accordance with the Guidelines for the Institutional Animal Care and Use Committee (IACUC) and approved by The First Affiliated Hospital of Guangxi University of Chinese Medicine (DW20170528-39).

### Enrichment of stem cell-like Huh7 spheres

Human HCC cells (Huh7) were purchased from iCell (Shanghai, China). First, the Huh7 cells were cultured in high glucose DMEM medium (iCell, Shanghai, China) supplemented with 10% fetal bovine serum (FBS) at 37 °C in a humidified incubator with 5% (v/v) CO_2_. After digestion and centrifugation, Huh7 cells were resuspended with stem cell conditioned culture medium [low glucose DMEM-F12 medium containing B27 (1:50), 0.4% BSA, EGF (20 ng/ml) and bFGF (20 ng/ml)]. Then, the cells were plated into 24-well ultra-low attachment plates with serum-free stem cell conditioned culture medium to form the spheres. The spheres were collected after 8 days of cell culture. Sphere-like cells were trypsinized into single cell suspension for follow-up experiments.

### HCC stem cell sorting

The supernatant of Huh7 cells was abandoned and cells were washed in PBS for twice before digested with 0.25% pancreatin. After that, cells were washed in PBS again and then filtered through 40 μm mesh for cell counting. Cell density was adjusted to 5 × 10^6^/ml and transferred into a FACS BD tube with 2 ml for each tube. Then 80 μl FACS sorting buffering was added into each tube for cell suspension and then the cells were added with 20 μl Fc R block buffer for incubation of 10 min. Cells were incubated with fluorescent antibody at 4 °C for 30 min before washing in 2 ml FACS sorting buffer. Cell sorting was performed in flow cytometry (BD, CA, USA) after 2 ml FACS sorting buffer was added.

### Preparation of heterotopic transplanted tumor

Heterotopic transplanted tumors were produced by injecting 100 µL of liver cancer stem cell suspensions subcutaneously into the nude mice. Orthotopic transplantation was performed when the subcutaneous tumor grew to 1 cm. Before inoculation, the tissues were obtained in sterile condition and put into saline solution to remove the surrounding connective tissues. Samples were cut into 1 × 1 mm fragments for use.

### Nude mice orthotopic tumor xenograft model

The mice were anesthetized with intraperitoneal injection of chloral hydrate at a dose of 350 mg/kg and then underwent laparotomy with a 1 cm transverse incision. The left lobe of the liver was exposed and a part of the liver surface was mechanically damaged with scissors. Orthotopic implantation model was established by anchoring the tumor fragments to the left hepatic lobe. The model establishment takes 4 weeks, after which corresponding treatments were performed in mice in each group.

No treatment was performed on mice in control group. Mice in BMSCs group were slowly injected with 1 mL BMSCs (1 × 10^6^  ells) through the hepatic portal vein fortnightly after model establishment with abdomen stitched [16, 17], while mice in BJJP group were daily administered intragastrically with 1.1 g/kg BJJP (Sinopharm Zhonglian, Wuhan, China) for continuously 4 weeks [18] after the model establishment. Mice in BJJP + BMSCs group were subjected to orthotopic tumor xenograft and received 1.1 g/kg BJJP daily by gavage and injection of BMSCs. Mice in BJJP + miR-140 inhibitor (Hycell, Wuhan, China) were subjected to orthotopic tumor xenograft and injected with miR-140 inhibitor once in 2 weeks through the caudal vein. Mice in BMSCs + miR-140 inhibitor group had same treatment as BMSCs group except for additional injection of miR-140 inhibitor once in 2 weeks through the caudal vein. Mice in BJJP + BMSCs + miR-140 inhibitor group received the same treatment as BJJP + BMSCs group except for additional injection of miR-140 inhibitor once in 2 weeks through the caudal vein. Mice in Model group were administrated with equal volume of normal saline. Mice in each group were sacrificed 8 weeks later, after that the liver tissues were fixed with 4% paraformaldehyde or stored in liquid nitrogen.

### Extraction and culture of BMSCs

BALB/c mice (male) were killed by pulling off cervical vertebrae and soaked in 75% ethanol for 10 min. Under aseptic condition, the femur and tibia were removed. After cutting off the epiphysis at both ends, bone marrow cells in the bone marrow cavity were washed out with low-glucose DMEM. The culture medium containing cell suspension was centrifuged at low speed for 5 min and the supernatant was discarded, and then re-suspended with low-glucose DMEM complete culture medium. The collected cells were cultured at 37 °C in a humidified incubator with 5% CO_2_. After incubation for 48 h, the non-adherent cells were removed. When cell fusion was 80–90%, the adherent cells were harvested by trypsin digestion.

### Differentiation ability of BMSCs

Adipogenic differentiation by oil red O staining: BMSCs (2 × 10^4^ cells/cm^2^) were inoculated into the six-well plates for cell fusion. Adipogenic differentiation was induced with adipogenic differentiation basal medium (MUCMX-90031, Cyagen, Guangzhou, China) when cell fusion reaches 100%. BMSCs were added with 2 ml of culture medium for OriCell BALB/C BMSCs culture medium (liquid A) after the original culture medium was removed. After adipogenic differentiation for 3 days, the liquid A in the six-well plates was removed and replaced with 2 ml of culture medium for OriCell BALB/C BMSCs culture medium (liquid B) for cell culture for 24 h. Then the liquid B was absorbed and replaced with liquid A. The inter-replacement of liquid A and liquid B was performed for 3–5 times (12–20 days), the BMSCs were cultured with liquid B for 4–7 day (refresh every 2–3 days) until the lipid droplets are big enough. The six-well plates were washed with 1 × PBS for 1–2 times and fixed with 2 ml 4% neutral formalin solution per well for 30 min. Then the formalin solution was removed and the six-well plates were washed with 1 × PBS for twice. After PBS wash, each well was added with 1 ml of oil red O dye liquor for 30 min (dye liquor formulate: oil red O: distill water = 3:2, filtered through the neutral filter paper). After the oil red O dye liquor was removed, the six-well plates were washed in 1 × PBS for 2–3 times and observed under a microscope.

Osteogenic differentiation and alizarin red staining: BMSCs of 2 × 10^4^ cell/cm^2^ were seeded into the gelatin-coated six-well plates. Osteogenic differentiation was induced with osteogenic differentiation culture medium (MUCMX-90021, Cyagen, Guangzhou, China) when cell fusion reaches 60–70%. After the culture medium was removed, the six-well plates were added with culture medium for 2 ml OriCell BALB/C BMSCs (refresh every 3 days) for 2–4 weeks. Alizarin red was used for cell staining. After staining, the culture medium was removed and the six-well plates were washed with 1 × PBS for 1–2 times, followed by fixation with 2 ml 4% neutral formalin solution per well for 30 min. Then the formalin solution was removed and the six-well plates were washed with 1 × PBS for twice. After PBS wash, each well was added with 1 ml of alizarin red. About 30 min later, the six-well plates was washed with 1 × PBS for 2–3 times and then observed under a microscope.

### Flow cytometry analysis

Flow cytometry analysis was conducted to evaluate the cell surface marker expressions of the CSCs. CSCs with various treatments were harvested and trypsinized into a single cell suspension. Subsequently, cells were incubated with fluorochrome-conjugated antibodies, including FITC-conjugated anti-human cluster of differentiation (CD) 24 (eBioscience, CA, USA), APC-conjugated anti-human CD133 (Biolegend, CA, USA) at 4 °C in the dark. The appropriated isotype-matched antibodies were used as negative controls. After 30-min incubation, the samples were centrifuged and washed with PBS for flow cytometry analysis. Similarly, the cell surface marker expressions of the BMSCs were also evaluated by flow cytometry. The conjugated-specific antibodies used were as follows: PE-conjugated anti-mouse CD14 (Biolegend, CA, USA), APC-conjugated anti-mouse CD34 (Biolegend, CA, USA), APC-conjugated anti-mouse CD45 (Biolegend, CA, USA), PE-conjugated anti-mouse CD44 (Biolegend, CA, USA), PE-conjugated anti-mouse CD73 (Biolegend, CA, USA), APC-conjugated anti-mouse CD105 (Biolegend, CA, USA), FITC-conjugated anti-mouse CD166 (abcam, CA, USA), FITC-conjugated anti-mouse CD29 (Biolegend, CA, USA). Cell analysis was performed using a Flow cytometer (BD Biosciences, CA, USA) and FolwJo software version 7.6 (FlowJo LLC, OR, USA).

### Histopathological analysis

The isolated liver tissues were fixed with 4% paraformaldehyde for 24 h. Subsequently, tissues were dehydrated with ethanol and embedded in paraffin. Paraffin-embedded tissues were serially cut into sections with a thickness of 4 μm and used for the following histopathological examination. Hematoxylin–eosin (H&E) staining was conducted to observe the histological changes. Van Gieson (VG) staining was performed to observe the collagen deposition. The sections were stained with hematoxylin and eosin (Aspen, Canada, USA) or VG solution according to the manufacturer's protocols. The histological morphology was observed under a light microscope (Olympus, Japan). Terminal deoxynucleotidyl transferase-mediated dUTP-biotin nick end labeling (TUNEL) assay was conducted to detect the cell apoptosis rate of liver tissues under the manufacturer's instructions. The cell nucleus was double-dyed using TUNEL kit and DAPI (Yeasen Biotech CO., Ltd., Shanghai, China). The TUNEL-positive cells were visualized using a fluorescence light microscope (Olympus, Japan).

### Immunofluorescence assay

Immunofluorescence assay was conducted to detect the levels of Ki67, a cell proliferation related protein. Briefly, sections were incubated with 3% H_2_O_2_ in the darkness and blocked with 5% BSA for 20 min. Subsequently, sections were incubated with anti-Ki67 primary antibody (Abcam, UK) at 4 °C overnight, followed by incubation of a fluorescence-labeled secondary antibody (Aspen, Canada, USA) for 50 min. Finally, after being counterstained with DAPI for 5 min, the sections were imaged using a fluorescence light microscope (Olympus, Japan). The images were analyzed by Image-Pro Plus 6.0 (Media Cybernetics).

### PCR analysis

qRT-PCR were performed to evaluate the mRNA expression levels of miR-140, CD24, CD133 and EpCAM in the liver tissues. Briefly, total RNA were extracted from tissues using Trizol TRIpure Total RNA Extraction Reagent (ELK Biotechnology, Wuhan, China) under the manufacturer's protocols. EntiLink™ 1st Strand cDNA Synthesis Kit (ELK Biotechnology, Wuhan, China) were applied for cDNA reverse-transcription. The EnTurbo™ SYBR Green PCR SuperMix Kit (ELK Biotechnology, Wuhan, China) was applied for the amplification of the synthesized cDNA. For CD24, CD133 and EpCAM, GAPDH served as the internal control. For miR-140, U6 served as the internal control. Relative expression of genes was determined using the 2^−ΔΔCt^ method. Primers used are listed in Table 1.

**Table 1:** Primer sequences used for qRT-PCR

Gene	Sequence (5′-3′)	
CD24	Forward	ACCCACGCAGATTTATTCCAG
	Reverse	CACGAAGAGACTGGCTGTTGAC
CD133	Forward	GCACTCTATACCAAAGCGTCAAG
	Reverse	GCACGATGCCACTTTCTCAC
EpCAM	Forward	GTGTGTGAACACTGCTGGGGT
	Reverse	CTGAAGTGCAGTCCGCAAACT
miR-140	Forward	GGGTAGAACCACGGCTCAAC
	Reverse	CTCAACTGGTGTCGTGGAGTC
GAPDH	Forward	CATCATCCCTGCCTCTACTGG
	Reverse	GTGGGTGTCGCTGTTGAAGTC
U6	Forward	CTCGCTTCGGCAGCACAT
	Reverse	AACGCTTCACGAATTTGCGT

### Western blot analysis

Western blots were performed to determine the protein expression levels of CD24, CD133 and EpCAM in the liver tissues. Briefly, protein lysates were extracted from the liver tissues using RIPA Lysis Buffer (Aspen, Canada, USA) supplemented with the protease inhibitor cocktail (Roche, Basel, Switzerland). Protein concentration was determined by BCA protein kit (Aspen, Canada, USA). The protein samples were separated with 10% SDS-PAGE and transferred to PVDF membrane. Then, the membrane were incubated with the primary antibodies overnight at 4 °C, followed by the corresponding horseradish peroxidase conjugated secondary antibodies at room temperature for 30 min. Finally, the membrane was developed with the enhanced chemiluminescence (ECL) substrate solution kit (Aspen, Canada, USA) for 1 min. The targeted protein bands were analyzed using with AlphaEaseFC software for gray scale value. GAPDH was used as the internal control. Primary antibodies used in this study are listed in Table 2.

**Table 2:** Detailed information for antibodies used for western blot

Antibody	Species	Manufacturer	Category no	Dilution ratio
GAPDH	Rabbit	Abcam	ab9485	1:10,000
CD24	Rabbit	Abcam	ab179821	1:500
CD133	Rabbit	Cell Signaling Technology	64,326	1:500
EpCAM	Rabbit	Abcam	ab223582	1:1000
Wnt3a	Rabbit	Abcam	ab219412	1:500
β-catenin	Rabbit	Abcam	ab32572	1:500

### Statistical analysis

All experiments were performed in triplicate. All data were presented as the mean ± standard deviation (SD). Statistical differences for multiple groups were conducted with One-way ANOVA and MANOVA using the GraphPad Prism 7.00 software (GraphPad Software, Inc.). Value of *P* < 0.05 was considered statistically significant.

## Results

### Identifications of CSCs and BMSCs

**Fig. 1:**
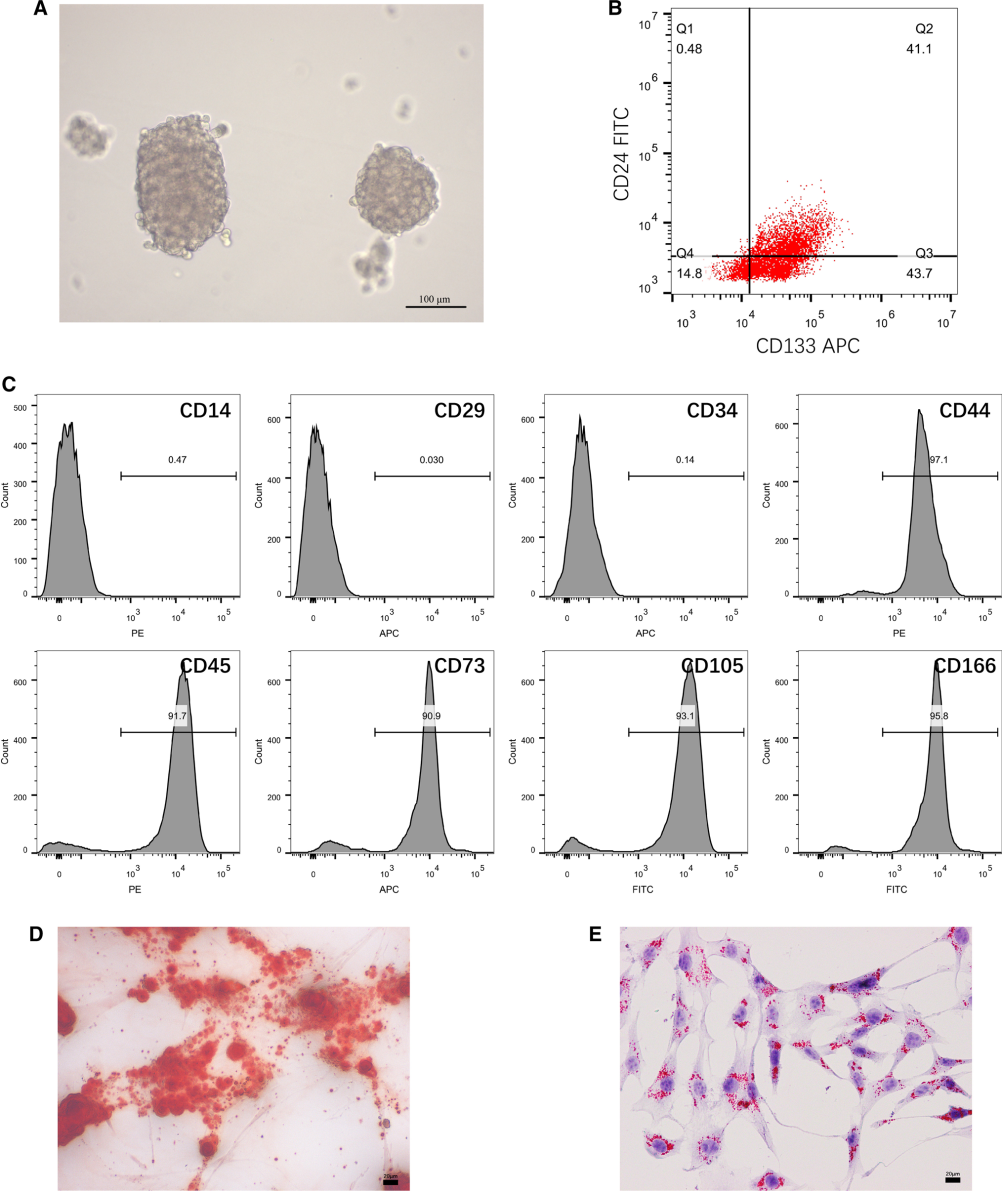
Identification of CSCs and BMSCs. **A** morphology of CSCs spheres; **B** flow cytometry was used to measure the expressions of specific stem cell markers CD24 and CD133 in CSCs; **C** the expressions of BMSC markers CD44, CD73, CD105, CD166, CD29,CD14, CD34 and CD45 were also measured by flow cytometry in isolated BMSCs. The adipogenic differentiation (**D**) and osteogenic differentiation (**E**) ability of isolated BMSCs was determined by oil red O staining and alizarin red staining, respectively. *CSCs* cancer stem cells, *BMSCs* bone mesenchymal stem cells

Huh7 cells were cultured with stem cell conditioned medium and formed large spheres after 8 days (Fig. 1A). CSCs enriched from Huh7 cells were confirmed by the expressions of specific stem cell markers CD24 and CD133 (Fig. 1B). As shown in Fig. 1C, the successfully extraction of BMSCs were confirmed by the positive expression of CD44, CD73, CD105, CD166 and CD29, and the negative expression of CD14, CD34 and CD45. The isolated BMSCs are capable of adipogenic differentiation and osteogenic differentiation (Fig. 1D, E), indicating the isolated BMSCs has differentiation ability. Collectively, the CSCs and BMSCs were successfully isolated and are qualified for later experiments.

### Combination of BJJP and BMSCs attenuates liver injury in HCC tissues

**Fig. 2:**
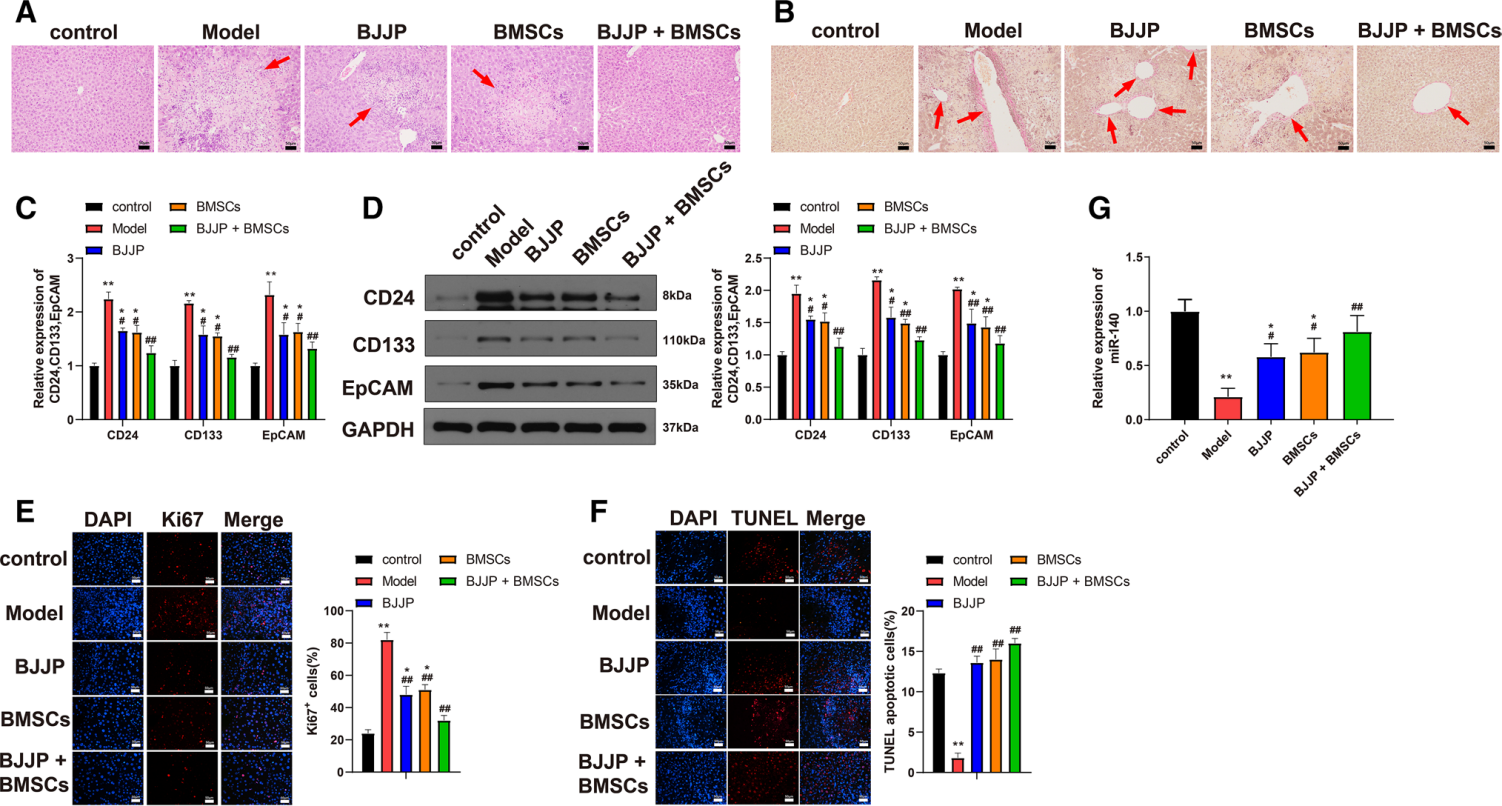
Combiation use of BJJP and BMSCs could enhance the proliferation of BMSCs and therefore ameliorate the liver injury in HCC. In vivo HCC mouse model was established to explore the protective role of BJJP and BMSCs in liver tissues. H&E (**A**) and VG (**B**) staining was performed to determine the liver tissue injury. RT-qPCR (**C**) and western blot (**D**) were used to detect the expressions of CD24, CD133 and EpCAM. Meanwhile, cell proliferation ability was assessed by measuring the expression of Ki67 (**E**). Cell apoptosis was determined by TUNEL staining (**F**). The expression of miR-140 was measured by RT-PCR (**G**). *N* = 5, *, vs control group, *P* < 0.05; **, vs control group, *P* < 0.01; #, vs Model group, P < 0.05; ##, vs Model group, *P* < 0.01. *HCC* hepatocellular carcinoma, *BMSCs* bone mesenchymal stem cells, *H&E* hematoxylin–eosin, *VG* Van Gieson, *BJJP* Bie Jia Jian pill

In vivo HCC model was established in mice (Supplemented figure S), which was identified through H&E and VG staining. RT-qPCR and western blot were applied to detect the expressions of CSC biomarkers, while immunofluorescence and TUNEL staining was used for Ki67 expression and cell apoptosis, respectively. The observation on liver tissues after H&E and VG staining showed that tissues of mice in Model group had noticeable necrosis, cell infiltration and fibrosis in comparison to that in control group (Fig. 2A, B). Meanwhile, elevated expressions of CD24, CD133, EpCAM (Fig. 2C, D  *P*< 0.01) and Ki67 (Fig. 2E  *P*< 0.01), as well as decreased cell apoptosis (Fig. 2F,  *P*< 0.01) were also found in Model group when compared with those in control group. Those results showed that in vivo HCC model was successfully established and qualified for following experiments.

To better elucidate the role of BJJP and BMSCs in HCC, the effect of BJJP + BMSCs, BJJP or BMSCs alone was compared. The detection showed that BJJP + BMSCs, BJJP or BMSCs alone can ameliorate the tissue damages and cell proliferation, and up-regulate miR-140 expression in addition to suppressing the expressions of CD24, CD133 and EpCAM and increasing the cell apoptosis of tumor cell when respectively compared with BJJP group, BMSCs group or Model group (Fig. 2A–G, * P*< 0.05).

### BJJP and BMSCs attenuates liver injury through regulating miR-140

**Fig. 3:**
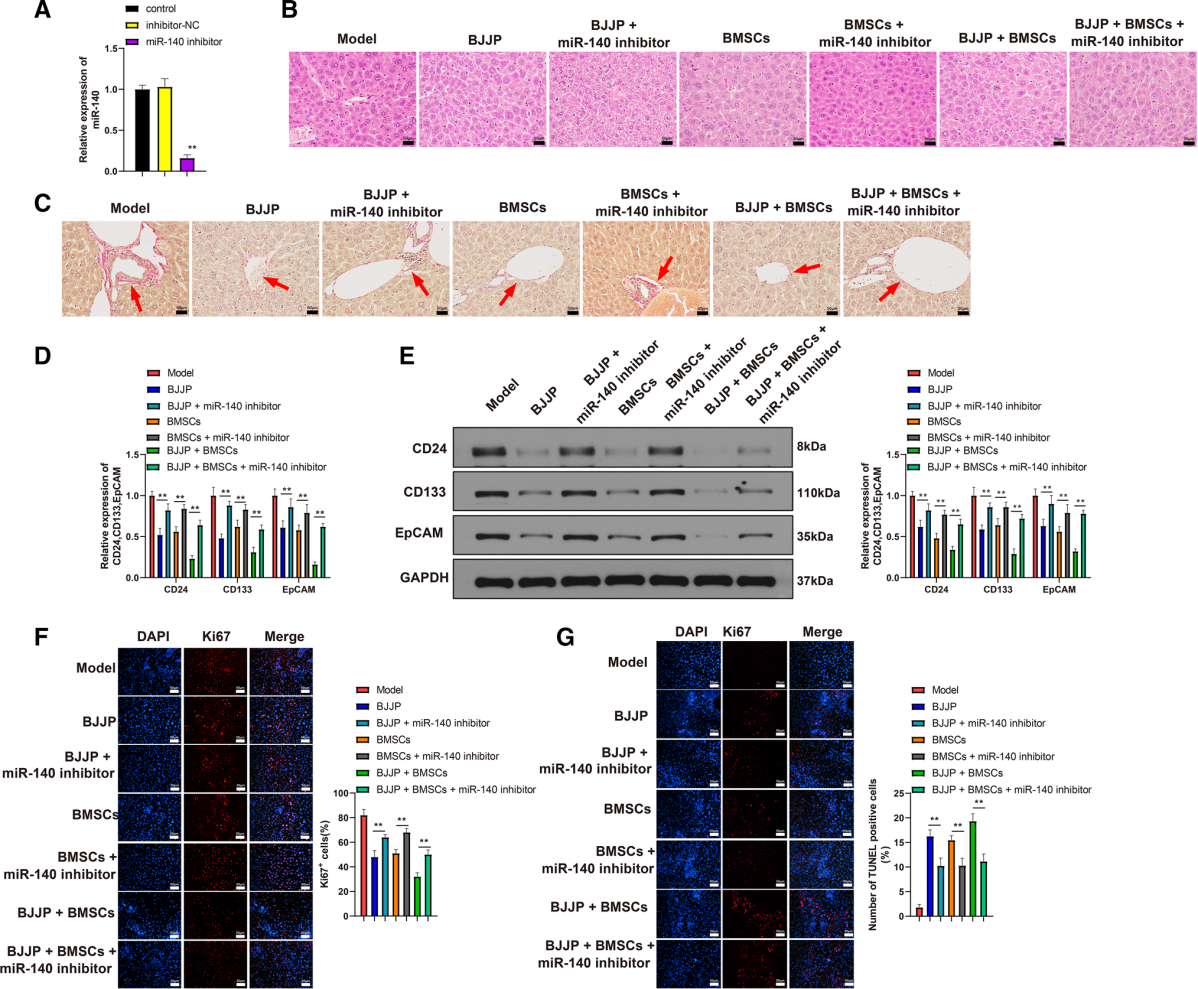
BJJP or/and BMSCs attenuates liver injury through up-regulating miR-140. The transfection efficiency of miR-140 was verified using RT-qPCR (**A**); The liver injury was determined by H&E staining (**B**) and VG staining (**C**); The expressions of CD24, CD133 and EpCAM were determined by RT-qPCR (**D**) and Western blot (**E**). Immunofluorescence was applied to detect the expression of Ki67 (**F**). Cell apoptosis was measured by TUNEL (**G**). *N* = 5, *P < 0.05; **P < 0.01; *BMSCs* bone mesenchymal stem cells, *H&E* hematoxylin–eosin, *VG* Van Gieson, *BJJP* Bie Jia Jian pill

Data supported the implication of miR-140 in the initiation and development of HCC. To verify whether BJJP and BMSCs regulates miR-140 in liver tissues, we first detected the expression of miR-140 in the liver of mice. The detection showed that compared with control group, the expression of miR-140 was decreased in Model group. Increased expression of miR-140 was found in BJJP and BMSCs groups when compared with Model group, while highest expression was found in BJJP + BMSCs group (Fig. 3A, *P*<0.05). Those data supported that BJJP and BMSCs can up-regulate miR-140 expression in the liver tissues.

To further determine whether BJJP and BMSCs exerts protective effect on liver tissues through regulating miR-140, we applied tail vein injection of miR-140 inhibitor into the mice. Measurement on miR-140 expression showed that miR-140 inhibitor could significantly suppress the expression of miR-140 (Fig. 3B, *P*<0.05). Treatment of BJJP or BMSCs in mouse showed that BJJP + miR-140 inhibitor group, BMSCs + miR-140 inhibitor group and BJJP + BMSCs + miR-140 inhibitor group had deteriorate necrosis, inflammatory infiltration, fibrosis proliferation and deposition in respectively comparison to those in BJJP group, BMSCs group and BJJP + BMSCs group (Fig. 3C–G, *P*<0.05). In addition to that, inhibition on miR-140 also leads to elevated expressions of CD24, CD133, EpCAM and Ki67, as well as suppressed cell apoptosis. Collectively, BJJP or/and BMSCs attenuate the injury in liver tissues through regulating miR-140.

### BJJP and BMSCs suppress Wnt/β-catenin signal pathway in CSCs of HCC

**Fig. 4:**
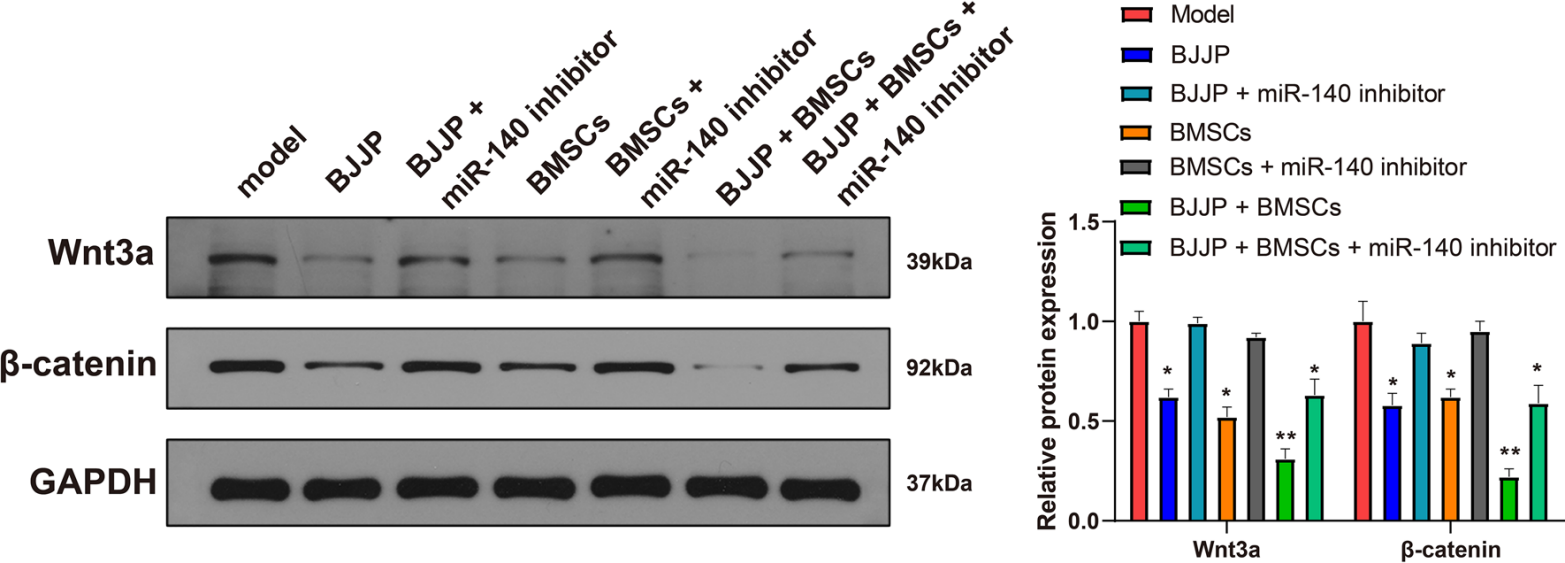
Combination of BJJP and BMSCs could suppress the Wnt/β-catenin signal pathway in CSCs in HCC model. Western blot was applied to measure the expressions of Wnt3a and β-catenin in Wnt/β-catenin signal pathway. *N* = 5, *P < 0.05; **P < 0.01; BJJP, Bie Jia Jian pill; BMSCs, bone mesenchymal stem cells; HCC, hepatocellular carcinoma; CSCs, cancer stem cells

Wnt/β-catenin signal pathway is vital for the regeneration and regulation of CSCs [19]. In this regard, we further explored the involvement of Wnt/β-catenin signal pathway in protective role of BJJP and BMSCs in HCC. Detection on the key protein of Wnt/β-catenin signal pathway, Wnt3a and β-catenin showed that the expressions of nt3a and β-catenin were suppressed in BJJP, BMSCs and BJJP + BMSCs groups, respectively compared to Model group, and BJJP or BMSCs group (Fig. 4, *P* < 0.05). However, inhibition on miR-140 in turn elevated the expressions of Wnt3a and β-catenin (BJJP + BMSCs + miR-140 inhibitor group vs BJJP + miR-140 inhibitor group or BMSCs + miR-140 inhibitor group, *P* < 0.05). Collectively, BJJP and BMSCs could regulate miR-140 to suppress the activation of Wnt/β-catenin signal pathway in HCC.

## Discussion

In this study, we first extracted CSCs from Huh7 cells to establish an orthotopic HCC xenograft model, from which the liver tissues were collected for extraction of BMSCs. The extracted BMSCs were collected for determination for cell proliferation and apoptosis of CSCs in mouse models. Meanwhile, BJJP was also fed to in vivo models to observe its effect on tumor progression. Taken together, our results showed that both BMSCs and BJJP could attenuate the disease progression of HCC in mice, while the combination used of BMSCs and BJJP had better therapeutic effect on suppressing tumor development than BMSCs or BJJP alone.

CSCs are considered to be an important subpopulation of cancer tissues, which are closely associated with tumor progression, recurrence and metastasis [20]. CD24, CD133 and EpCAM have been identified as the CSCs cell-surface markers in the HCC [21]. In this study, flow cytometry was applied to detect the expressions of CD24 and CD133 in CSCs developed by Huh7 cells. We found that BJJP significantly reduced the expressions of CD24, CD133 and EpCAM in the liver cancer tissues, indicating BJJP could suppress the properties of CSCs in HCC. Further experiments by H&E, TUNEL and Ki67 staining showed that BJJP treatment ameliorated liver injury and inhibited Ki67 expressions in addition to promoting cell apoptosis. These findings together revealed the protective role of BJJP in HCC.

BMSCs are a kind of cells with multi-differentiate potentials, which can differentiate into cells of mesenchymal or other lineage, and therefore have been considered as an attractive candidate for stem cell-based treatment [22]. Studies have shown that MSCs can repair liver damage after hepatogenic differentiation and have anti-inflammatory, anti-fibrotic and anti-apoptotic effects on hepatocytes [6, 23]. In this study, BMSCs was proved to improve necrosis, infiltration of inflammatory cells and fibrotic hyperplasia in the liver cancer model. In addition, the treatment of BMSCs resulted in a significant increase of apoptotic cells in the liver cancer tissues. Ki67 is an index of cell proliferation. The number of Ki67-positive cells was markedly decreased in the liver cancer tissues in response to BMSCs treatment, indicating that BMSCs strongly accelerate liver cancer cell apoptosis while suppressing cell proliferation. These results indicate that the protective effects of BMSCs in HCC may be related to its anti-apoptotic function. Our result was supported by previous findings which showed that MSC had the potential to control metastasis of HCC cells [24].

The protective role of both BMSCs and BJJP in HCC models was reported in previous study and a recent study declared the mechanism herein involved the activation of Wnt/β-catenin Signaling Pathway [25], a comprehensive understanding on the mechanism involved was far from being fully elucidated. Considering the tumor suppressing role of miR-140 in HCC [11, 12], we further explore the possible role of miR-140 in BMSCs and BJJP regulated HCC models. The detection showed that miR-140 was remarkably reduced in HCC model, which is consistent with the previous studies. Moreover, administration of BJJP or BMSCs alone and co-treatment of BJJP and BMSCs significantly increase miR-140 expression in the liver cancer tissues. Inhibition on miR-140 expression further reversed the protective effect of BJJP, BMSCs and BJJP + BMSCs. Those results proved that both BJJP and BMSCs attenuate liver injury in HCC models through regulating miR-140 expression. Further detection on the downstream signal pathway of miR-140 showed that Wnt/β-catenin signal pathway was suppressed in response to BJJP or BMSCs treatment, while suppression on miR-140 could reverse the suppression of BJJP and BMSCs in Wnt/β-catenin signal pathway. The regulation of miR-140 on Wnt/β-catenin signal pathway can be found in both carcinoma tissues and other diseases, including lung cancer, malignant melanoma, nasopharynx cancer and sepsis [26–29]. Taken together, those results showed that BJJP and BMSCs could elevate miR-140 expression, which in turn could suppress the activation of Wnt/β-catenin signal pathway.

The major highlights in this study is that we also found that combination use of BJJP and BMSCs had better therapeutic effect than BJJP or BMSCs alone and could further elevate miR-140 expression in HCC models, which suggested the potential of BJJP and BMSCs treatment together in controlling HCC progression. However, evidence in this study can only prove the involvement of miR-140 and Wnt/β-catenin signal pathway in protective effect of BJJP and BMSCs. The possible effect or interaction of BJJP and BMSCs in HCC remains to be further determined and will be considered as our future goal.

In conclusion, we demonstrated that the BJJP and BMSCs alone or combination could inhibit HCC progression by up-regulating miR-140 expression and suppressing Wnt/β-catenin signal pathway. Our study provides a preliminary understanding of the role of BJJP and BMSCs in the treatment of HCC.

## Supplementary Information

Below is the link to the electronic supplementary material.

Supplementary file1 (TIF 62949 KB)
