# Supplementary figures and images for: Bie Jia Jian pill enhances the amelioration of bone mesenchymal stem cells on hepatocellular carcinoma progression
Source: J Nat Med. 2021 Jul 23;76(1):49–58. doi: 10.1007/s11418-021-01548-4 (PMC8732910; doi:10.1007/s11418-021-01548-4)

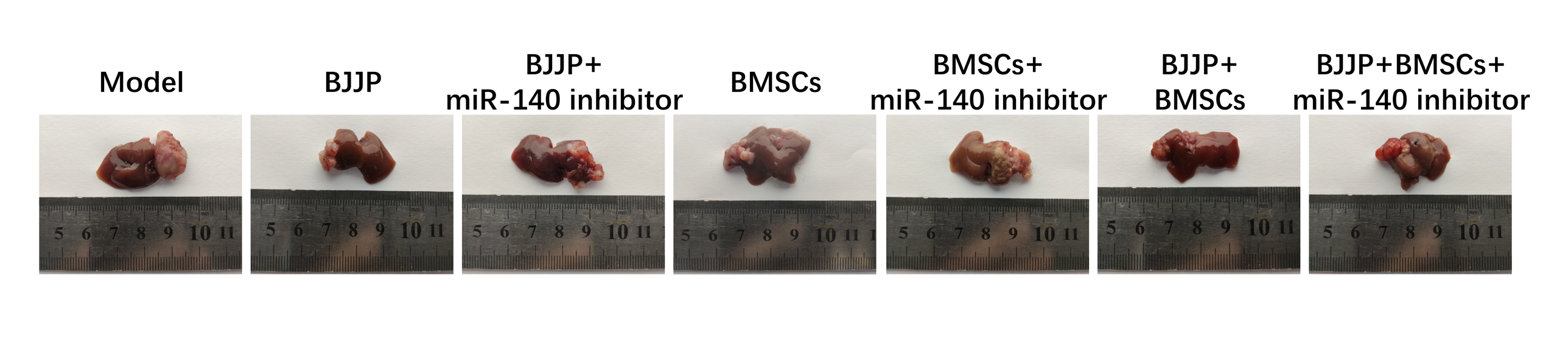

Supplement: Supplementary file 1 — Supplementary file1 (TIF 62949 KB) [file 11418_2021_1548_MOESM1_ESM.tif]
